# Ion Interference Therapy of Tumors Based on Inorganic Nanoparticles

**DOI:** 10.3390/bios12020100

**Published:** 2022-02-06

**Authors:** Yongjie Chi, Peng Sun, Yuan Gao, Jing Zhang, Lianyan Wang

**Affiliations:** 1 Institute of Process Engineering, Chinese Academy of Sciences, Beijing 100190, China; chiyongjie21@ipe.ac.cn (Y.C.); sunpeng23333@163.com (P.S.); m18342201571@163.com (Y.G.); zhangjin@ipe.ac.cn (J.Z.); 2 University of Chinese Academy of Sciences, Beijing 100190, China; 3 College of Biological Science and Technology, Shenyang Agricultural University, Shenyang 110866, China; 4 Key Laboratory of Forest Plant Ecology, Ministry of Education, College of Chemistry, Chemistry Engineering and Resource Utilization, Northeast Forestry University, Harbin 150040, China

**Keywords:** ion interference therapy, cancer, ions, inorganic nanoparticles

## Abstract

As an essential substance for cell life activities, ions play an important role in controlling cell osmotic pressure balance, intracellular acid–base balance, signal transmission, biocatalysis and so on. The imbalance of ion homeostasis in cells will seriously affect the activities of cells, cause irreversible damage to cells or induce cell death. Therefore, artificially interfering with the ion homeostasis in tumor cells has become a new means to inhibit the proliferation of tumor cells. This treatment is called ion interference therapy (IIT). Although some molecular carriers of ions have been developed for intracellular ion delivery, inorganic nanoparticles are widely used in ion interference therapy because of their higher ion delivery ability and higher biocompatibility compared with molecular carriers. This article reviewed the recent development of IIT based on inorganic nanoparticles and summarized the advantages and disadvantages of this treatment and the challenges of future development, hoping to provide a reference for future research.

## 1. Introduction

Cancer is still one of the leading causes of death globally [1]. Chemotherapy, as one of the main cancer treatment methods, is widely used especially for advanced cancers in clinic [2]. In order to reduce the side effects of chemotherapy, such as systemic toxicity, multifarious nanoparticles were constructed as drug carriers to achieve targeted and sustained cancer therapy [3]. However, in such a pattern of treatment, some inherent properties of inorganic nanocarriers, such as the properties of absorbing hydrogen ions and releasing metal or nonmetal ions, sometimes seem to be ignored [4]. In the latest research, the released ions in different intracellular environments by inorganic nanoparticles have made a great contribution to inhibiting the activity of cancer cells and enhancing the therapeutic effect of chemotherapy. Therefore, the research based on the ion release of inorganic nanoparticles and its anticancer mechanism has broad prospects. Meanwhile, combining this with immunotherapy to comprehensively improve the anti-cancer ability will be a new option in the future.

Ions exist widely in the human body and have always been the focus of research due to their participation in various life activities, including osmotic pressure, the acid–base balance, catalytic and signal pathway activation, the protein and enzyme composition, targeting biomolecules and so on [5]. The abnormal distribution or specific accumulation of some metal or non-metal ions in cells cause irreversible damage to cells or activate cytotoxicity-related biochemical reactions to induce cell death, which provides a new method for tumor therapy, namely ion interference therapy [6]. However, the application of some small molecule carriers frequently used for ion interference therapy, often encounters obstacles, such as short internal circulation, dose-dependent toxicity, poor specific recognition ability and a low dose of ion release, which limits the effect of cancer treatment [7]. Based on this, inorganic nanoparticles such as Ca_2_O, ZnO, CaCO_3_, BPS (black phosphorus) and NaCl have been constructed for ion interference treatment, with the advantages of long internal circulation, efficient inhibition of tumor development, high biocompatibility and strong specific recognition ability (Table 1) [4,8,9,10,11]. Because of these advantages, ion interference therapy based on inorganic nanoparticles, as an emerging cancer treatment, represents strong competition for traditional cancer treatment.

Another major finding about ion interference therapy is that it can enhance the effect of antitumor immunity. Normally, the human immune system can recognize and remove cancer cells. Daniel S. Chen and IRA Mellman divided the fight between the immune system and cancer cells into seven stages in 2013, collectively referred to as the tumor immune cycle. The seven stages are: (1) Tumor cell death releases antigen; (2) Antigen presentation; (3) Start and activate T cells; (4) Cytotoxic T lymphocyte (CTL) is transported to tumor tissue through blood circulation; (5) Cytotoxic T lymphocyte infiltration in tumor tissue; (6) Cytotoxic T lymphocytes recognize tumor cells; (7) Kill cancer cells and finally return to the first step [12]. If the immune process is abnormal at any stage in the cycle, or the cancer cells themselves take some strategy to inhibit the immunity of a link, it can lead to immune escape [13]. In the process of the tumor immune cycle, inducing immunogenic death of tumor cells which can produce a large number of antigens is an important step, so it has been an important focus of immunotherapy for many years. Some traditional methods of cancer treatment have been proved to stimulate the immunogenic death of tumor cells, such as chemotherapy (adriamycin, oxaliplatin, etc.), radiotherapy, etc. Recently, new studies have shown that interfering with the balance of certain ions in the intracellular environment can also lead to the immunogenic death of cells, such as pyroptosis, which was characterized by the continuous swelling of cancer cells until the rupture of the cell membrane and the release of a large number of cell contents, with no obvious side effects compared with chemotherapy and radiotherapy [11]. The immunogenic death of cancer cells finally promotes the antigen release and presentation process, so as to enhance the patient’s own ability to clear cancer cells and immunity. Some ions can also accumulate in immunocytes, such as dendritic cells (DCs), so as to promote the activation of immunocytes and the efficiency of antigen presentation, and finally realize the enhancement of immunity [14]. Therefore, using ion interference therapy based on inorganic nanoparticles to activate and promote the patient’s immune system to achieve efficient cancer treatment is a reliable means. At the same time, combined it with new immunotherapy methods, such as immune checkpoint inhibitor therapy, will also produce better anti-cancer effects. Consequently, the combination of ion interference therapy and tumor immunotherapy may be a valuable route for humans to fight cancer in the future.

Although chemotherapy and immunotherapy have achieved great success in anticancer, there are still some complex troubles in the processes of chemotherapy and tumor immune cycle, which may eventually lead to a low therapeutic effect. In recent years, the research into ion interference therapy based on inorganic nanoparticles has been increasingly explored due to its high efficiency in inhibiting tumor cell proliferation and its ability to promote antitumor immunity. Therefore, making full use of and highlighting the various excellent properties of ion released by nanoparticles to achieve multitherapy is a matter of concern. In this review, we discuss ion interference therapy and ion-related tumor immunotherapy based on inorganic nanoparticles and explore the future perspectives of ion interference therapy in the treatment of cancer.

## 2. Ion Interference Therapy

**Figure 1:**
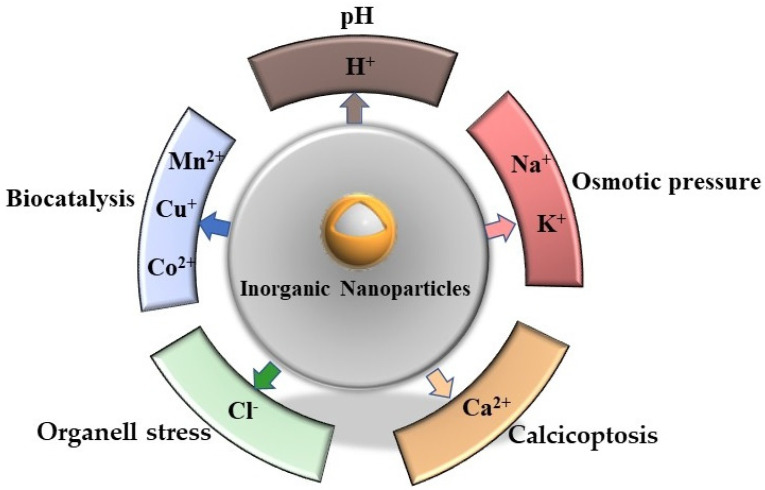
A schematic overview of ion interference antitumor therapy.

Intracellular ions strictly control the life process of cells, such as membrane potential regulation, osmotic balance, protein synthesis, signal transduction etc. [15]. The basic idea of killing cancer cells by regulating the homeostasis of some ions in cells has long been applied in the field of cancer therapy, and has achieved breakthrough results, such as “ferroptosis“. In order to pass through the cell membrane barrier favorably, Fe^2+^ ions which are loaded on nanoparticles enter into tumor cells by endocytosis. Then, ferrous ions are converted into Fe^3+^ via the Fenton reaction upon the induction of the intracellular specific microenvironment and finally stimulate cells to produce a large number of reactive oxygen species (ROS) and lipid peroxides, resulting in the death of cancer cells [16,17]. This principle was first classified as “regulated cell death“ (RC) because it is highly dependent on Fe^2+^ and regulated by Fe^2+^ [17]. However, with the in-depth study of intracellular ion action and cell signal pathways, Wenbo Bu et al. summarized the research on promoting cancer cell death based on ion induction or regulation as “ion interference therapy” [6]. In recent years, the research into ion interference therapy has attracted much attention. Some metal or non-metal ions (such as H^+^, Na^+^, K^+^, Cl^−^, Ca^2+^, Mn^2+^, Cu^+^) have been applied and have good effects in inhibiting tumors (Figure 1).

### 2.1. Protons in Tumor Cells

H^+^ ions are widely distributed inside and outside cells to regulate the survival microenvironment of cells and the acid–base balance in cells. In particular, the acidic organelle in cells, the lysosome, as a proton store, is the center responsible for degradation, nutrient sensing and immunity [18]. At the same time, lysosomes are also closely related to the growth, proliferation and migration of tumor cells [19]. Targeting lysosomes to achieve H^+^-mediated ion interference therapy is also an effective means to treat cancers.

**Figure 2:**
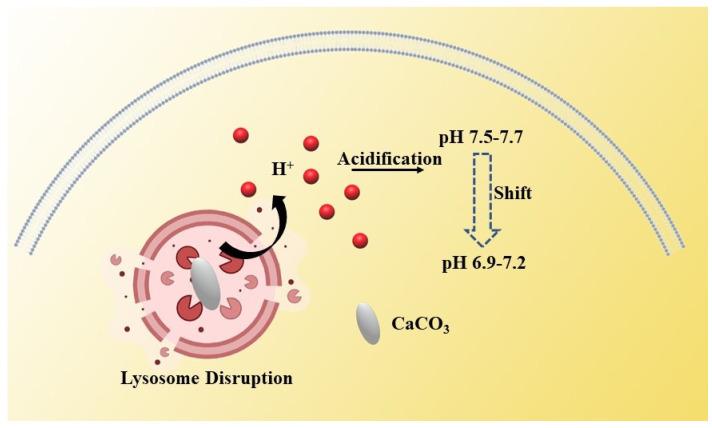
Schematic illustration of H^+^—induced antitumor therapy. The expansion of CaCO_3_ nanoparticles will lead to lysosome expansion and the extrusion of a large amount of H^+^, resulting in intracellular acidification.

Under normal conditions, to avoid cellular acidosis, cells maintain a balanced internal and external pH, usually 7.2–7.4 inside normal cells and 7.6 inside tumor cells [20]. This is due to the efflux of hydrogen ions in tumor cells, resulting in the pH of cytoplasm inside the tumor cells being higher than that of normal cells, while the pH outside the tumor cells is lower than that of normal cells—that is, the so-called acidic microenvironment. When nanoparticles gain access into the lysosome, the degradation or expansion of nanoparticles can cause lysosome swelling and the extrusion of abundant H^+^ into the cytoplasm, which can induce intracellular acidification (Figure 2) [21]. The intracellular abnormal acidic environment will seriously affect the activity of cells. Therefore, we can interfere with the proliferation of tumor cells by regulating the homeostasis of intracellular hydrogen ions.

Nano-CaCO_3_ as one kind of ideal vector with high biocompatibility, and can also be used to interfere with the intracellular homeostasis of lysosome hydrogen ions by utilizing the acid response of calcium carbonate, which was so called “lysosome bombs”, according to Chenxu Zhang et al. [20]. The nano-vaterite calcium carbonate was coated with disulfide-cross-linked sodium alginate (DSA) and loaded with doxorubicin. When the nanoparticles are swallowed by tumor cells, the DSA on the surface of nano-CaCO_3_ degrades in response to glutathione. Then, the exposed calcium carbonate particles enter the lysosome inside the tumor cell. Due to the expansion of nano-CaCO_3_ in the lysosome, lysosome membrane will rupture rapidly and release acidic inclusions. The rapid change in intracellular pH makes tumor cells more sensitive to chemotherapeutic drugs, and finally accelerates the death of cancer cells. It can be seen that it is feasible to realize cancer treatment by interfering with the pH environment inside tumor cells by inorganic nanoparticles. However, there are still many places worth discussing and improving. Through a large number of experimental studies, although nano-calcium carbonate without modification can instantly change the pH environment of cytoplasm in cells, it is difficult to achieve a hydrogen ion interference treatment without relying on chemotherapeutic drugs. This may be due to the reaction between nano-calcium carbonate itself and hydrogen ions, which weakens the decline in pH in the cytoplasm. Secondly, the changes in the pH environment inside tumor cells caused by nanoparticles cannot always be maintained for a long time. When the self-regulatory ability of tumor cells is relatively strong, the effect of this treatment modality is diminished. Therefore, how to design a novel inorganic nanoparticle that can continuously change the intracellular pH and realize a treatment modality based on hydrogen ion interference under conditions independent of other treatments such as chemotherapy will be a new research direction.

At the same time, according to the above research results, the tumor cell death caused by hydrogen ion interference therapy is likely to be a programmed death. Programmed cell death is likely to release more antigens, which can promote the presentation efficiency of antigens to dendritic cells, and finally enhance the anti-cancer immune performance. Therefore, combined with this characteristic, it has potential to explore the application of hydrogen ion interference therapy in immunotherapy.

### 2.2. Sodium and Potassium Ions in Tumor Cells

The plasma membranes of mammalian cells are not protected by a cell wall, as with plant cells. Therefore, when mammalian cells are under abnormal osmotic pressure, cells swell easily, resulting in cell membrane rupture and cell death [22]. Generally speaking, due to the existence of a sodium and potassium ion exchange pump on the cell membrane, the concentration of potassium ions in cells is higher than that outside cells, so as to maintain their normal structure and physiological activity [23]. Once the concentration gradient of these ions exceeds the limit of cell self-regulation, the osmotic pressure will change sharply, resulting in the destruction of the cytoskeleton, the reduction in cell activity, and even cell lysis [24]. Therefore, interference with ion balance can lead to effective antitumor therapy. Therefore, many Na^+^ and K^+^ ion carriers have been designed for ion interference therapy of tumors, including molecular carriers and inorganic nanoparticle carriers.

Recently, a molecular carrier, helical polypeptide-based potassium ionophores was designed by Daeyong Lee et al. to achieve transmembrane transport of K^+^ [25]. This molecular carrier can easily carry potassium ions through the cell membrane to the outside of tumor cells. When this carrier transports a large amount of K^+^ outside the cell, the balance of the K^+^ concentration in the cell is broken. The imbalance of intracellular potassium ion levels results in the strong activation of the unfolded protein response (UPR) in the endoplasmic reticulum (ER). The stressed ER leads to oxidative conditions by overproducing ROS, thereby damaging the mitochondria and activating apoptosis signaling. Therefore, K^+^ has been proved to have a significant effect in inducing tumor cell death. In addition to the method of inputting relevant ions into cells in the form of carrier, it can also be realized by blocking relevant ion channels on cell membrane. For example, Lin Zhu, Ge Wang et al. used chondroitin sulfate to mineralize a layer of calcium carbonate membrane on the surface of cancer cell membrane, which will seriously affect the function of the Na^+^/K^+^ ion channel on the surface of the cell membrane and induce the imbalance of ion concentration in cells, so as to promote the death of cancer cells [26]. Although this is a very effective method for ion interference, the number of ions that can be transported by molecular carriers is very low. Therefore, new and efficient ion transport methods have become the focus of attention, and inorganic nanoparticles happen to show more advantages in this regard.

**Figure 3:**
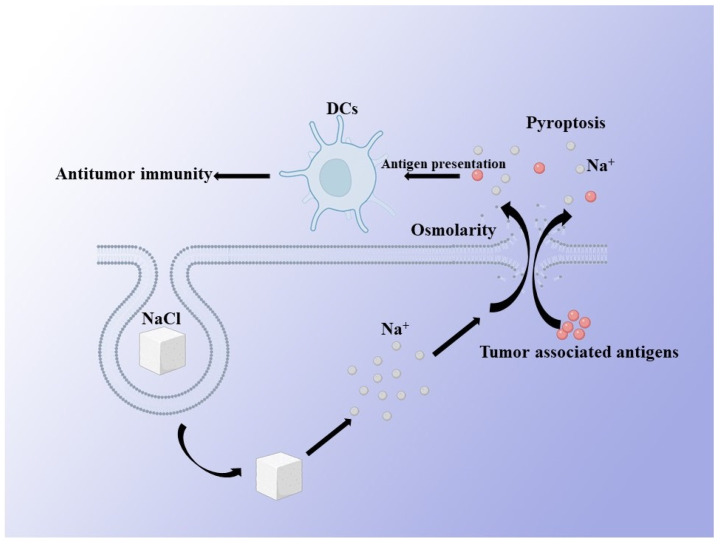
Schematic illustration of Na^+^—induced antitumor therapy. A large number of Na^+^ ions being released by NaCl nanoparticles in cancer cells leads to a change in cell osmotic pressure and further induces pyroptosis of cancer cells. Pyroptosis can promote the presentation efficiency of tumor-associated antigens to DCs, thus enhancing the antitumor immunity.

Compared with the molecular ion transporters and ion channel blockers, inorganic nanoparticles that can degrade in cells can achieve larger and more efficient ion transport, so as to interfere with the ion homeostasis in cells. Wen Jiang, Lei Yin et al. synthesized nanoparticles of NaCl (SCNPs), which are stable in the body fluid environment, through a microemulsion reaction. The reaction took place in a hexane/ethanol mixed solvent, with sodium oleate and molybdenum chloride as sodium and chloride precursors, and oleylamine as a surfactant. The as-synthesized SCNPs are hydrophobic because of the oleylamine coating. Additionally, they modified the SCNPs with PEGylated phospholipid. After the tumor cells engulfed the NaCl nanoparticles, the degradation of NaCl nanoparticles caused the explosive increase in the intracellular sodium ion concentration, resulting in the imbalance of intracellular and extracellular sodium ions, the change in cell osmotic pressure, and finally the death of tumor cells (Figure 3) [11]. Similarly, Yang Liu et al. designed a phospholipid-coated Na_2_S_2_O_8_ nanoparticle. They also realized the massive accumulation of sodium ions in tumor cells, resulting in the imbalance of osmotic pressure inside and outside the cells, and finally promoted the swelling and death of cells [27]. In their study, they also evaluated the synergistic effect of chemotherapy. They found that the therapeutic effect of chemotherapy could be significantly enhanced by interfering with the homeostasis of intracellular sodium ions [11,27]. Recently, biodegradable K_3_ZrF_7_:Yb/Er upconversion nanoparticles ZrNPs, which are similar to ion reservoirs, have been developed which can be dissolved inside cancer cells and release high amounts of K^+^ and [ZrF_7_]^3−^ ions, resulting a surge in intracellular osmolarity and homeostasis imbalance, according to Binbin Ding et al. [28]. Through biodegradable inorganic nanoparticles containing sodium and potassium, this can artificially regulate the internal and external osmotic balance of tumor cells, and finally significantly inhibit the proliferation of tumor cells. In these studies, it is worth noting that, compared with tumor cells, normal cells have higher tolerance to abnormal ion homeostasis. Therefore, these inorganic nanoparticles will not damage normal cells. Therefore, compared with traditional cancer treatment methods, such as chemotherapy, Na^+^- and K^+^-based ion interference therapy has higher biocompatibility and is not limited by drug resistance. In general, this treatment is expected to enter clinical trials. However, if better targeting effects can be provided, so that more nanoparticles can enter tumor cells, and a longer in vivo circulation cycle can be developed, as nano-NaCl has only a short cycle of 24 h, it will be more beneficial to the treatment of cancer.

At the same time, the pyroptosis which is induced by the imbalance of intracellular ion homeostasis is more noteworthy. Pyroptosis, also known as inflammatory necrosis, is a kind of programmed cell death which is characterized by the continuous expansion of cells until the rupture of the cell membrane, leading to the release of cell contents and then activating a strong inflammatory response [29]. Regardless of whether NaCl, Na_2_S_2_O_8_, or ZrNPs were used, all could induce pyroptosis, exhibiting superior antitumor immunity activity, as confirmed by enhanced dendritic cell (DC) maturity and the frequency of effector-memory T cells, as well as observably inhibiting tumor growth and pulmonary metastasis. Such research results show that Na^+^ and K^+^ ion interference therapy can greatly promote the patient’s own immunity, so as to improve the anti-cancer effect. This makes the advantages of ion interference therapy based on inorganic nanoparticles more obvious, but the relevant research cannot be limited to this. For example, whether the balance of sodium and potassium ions can also change the activity of immune cells, such as promoting the activation of dendritic cells, the phenotypic transformation of tumor associated macrophages from M2 to M1, the activation of T cells, etc., needs to be explored. Therefore, research into ion interference therapy in immunity needs to be further conducted, and ion interference therapy combined with immunotherapy may be a promising treatment in the future.

### 2.3. Calcicoptosis of Tumor Cells

Calcium is an important regulator of many cellular processes, such as muscle contraction, gene transcription, hormone release, neural signal transduction etc. [30]. In 2000, Michael R. Duchen summarized in detail the complex relationship between calcium ion signals and mitochondria [31]. On the one hand, the accumulation of Ca^2+^ in mitochondria can lead to the transient polarization of mitochondrial membrane potential. On the other hand, under the pathological conditions of intracellular calcium concentration overload, especially related to oxidative stress, the uptake of Ca^2+^ by mitochondria will trigger cell death. Based on this, in 2019, Maike Glitsch claimed that the regulation of calcium by related proteins or ion channels is expected to become another new method for tumor treatment [32]. It can be seen that calcium ions have broad research prospects in the field of tumor therapy [33].

More recently, given the importance of calcium ions in multiple cellular processes, calcium overload, characterized by an abnormal cytoplasm accumulation of free calcium ions (Ca^2+^), is a widely recognized cause of cell damage and even cell death in numerous cell types. This undesirable destructive process can become a new tool applicable to ion interference therapy. Hence, some calcium-based inorganic nanoparticles were constructed to achieve calcium ion interference therapy, such as CaO_2_, CaP and amorphous calcium carbonate.

**Figure 4:**
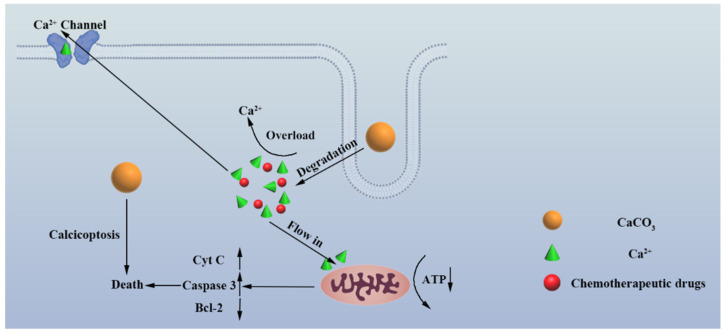
Schematic illustration of calcium overload-induced calcicoptosis. The overload of calcium ions in cancer cells promotes the damage of mitochondria, which induces specific calcium overload-induced cell death, which is called “calcicoptosis”.

Calcium-based inorganic NPs, which can mediate intracellular calcium homeostasis interference therapy, namely “calcicoptosis”, have attracted extensive attention (Figure 4). On the basis of the unique biological effects of Ca^2+^, Bu et al. demonstrated a highly efficient strategy for tumor therapy by utilizing pH-sensitive sodium hyaluronate-modified calcium peroxide NPs (SH-CaO_2_ NPs) [8]. Effective modification with sodium hyaluronate, which was performed alongside the nucleation and growth of the CaO_2_ NPs to confine the grain size, was achieved by the attraction between the negatively charged ions and positively charged nanocrystals. SH-CaO_2_ could slowly decompose into free Ca^2+^ and H_2_O_2_ in the acid tumoral microenvironment, leading to intracellular calcium overload and oxidative stress, resulting in the desensitization of calcium-related channels followed by an uncontrollable cellular accumulation of Ca^2+^ [8]. Overloaded calcium eventually leads to the decrease in mitochondrial membrane potential, mitochondrial damage and cell death. Notably, the killing effect is not limited to tumor types or hypoxic cells, and normal cells are more tolerant of the adverse influence of NPs than tumor cells. In 2021, Shi et al. reported a simple, yet versatile, tumor-targeting “calcium ion nanogenerator” (TCaNG) to reverse drug resistance by inducing intracellular Ca^2+^ bursting [34]. The TCaNG was prepared by loading the antitumor drug doxorubicin (DOX) into calcium phosphate (CaP) nanoparticles and then enveloping them with RGD peptide-decorated DSPE-PEG. Benefiting from the tumor vessel targeting effect of RGD, the TCaNG can be enriched in tumor tissues and internalized by tumor cells. Consequently, the TCaNG could induce Ca^2+^ bursting in acidic lysosomes of tumor cells and then reverse drug resistance to improve cancer treatment. Similarly, the work of Cheng Wang et al. confirmed that the excessive calcium ions produced by amorphous calcium carbonate initiated the apoptosis program and killed cancer cells in cooperation with chemotherapy by using phospholipid-modified amorphous calcium carbonate [7]. Due to the rapid degradation of amorphous calcium carbonate in tumor cells, a large number of calcium ions eventually lead to the death of tumor cells by damaging mitochondria. Yu Bin Dong’s team proved that Ca^2+^ overload and photodynamics can produce obvious synergistic killing effect by constructing a nanoscale covalent organic framework (NCOF)-based nanoagent, namely CaCO_3_@COF-BODIPY-2I@GAG, which is embedded with CaCO_3_ nanoparticles (NPs) and has a surface decorated with BODIPY-2I as a photosensitizer (PS) and glycosaminoglycan (GAG) targeting agent for CD44 receptors on the digestive tract tumor cells [35]. Xue Feng Yu et al.’s work significantly inhibited the proliferation of cancer cells through calcium overload by utilizing the prepared CaP mineralized black phosphorus material (CaBPs) [4]. CaBPs exhibit enhanced and selective anticancer bioactivity due to the improved pH-responsive degradation behavior and intracellular Ca^2+^ overloading in cancer cells. Furthermore, CaBPs specifically target mitochondria and cause structural damage, thus leading to mitochondria-mediated apoptosis in cancer cells. Jun Lin et al. emphasized the important role of calcium interference therapy of cuprous oxide nanoparticles coated with calcium carbonate [36]. With CaCO_3_ responsive to pH decomposition and Cu_2_O responsive to H_2_S sulfuration, Cu_2_O@CaCO_3_ can be turned “on” in the therapeutic mode by the colorectal TME. When the CaCO_3_ shell decomposes and releases calcium in acidic colorectal TME, excessive calcium accumulates in the cells, and finally cooperates with Cu_2_O degradation to stimulate the production of a large amount of reactive oxygen species (ROS), which promotes the death of cancer cells. Other calcium-containing nano-formulations were also developed for the purpose of cancer calcicoptosis, and the idea of “calcium-interference therapy” would potentially open up new opportunities for the development of antitumor strategies with high safety and precision.

Although the ion therapy based on calcicoptosis has achieved great success both in vitro anti-tumor experiments and in vivo anti-tumor experiments in mice, the anti-tumor effect of single intracellular calcium interference therapy does not seem to be so obvious. Therefore, calcicoptosis combined with other cancer treatments, such as photothermal therapy, chemokinetic therapy and immunotherapy, is a more effective means. At present, research on the effect of calcium on immunotherapy has also made some progress.

The acid-sensitive PEG-decorated calcium carbonate (CaCO_3_) nanoparticle incorporating curcumin, which was designed by Pan Zheng et al., can successfully induce calcium overload in the mitochondria in cells under ultrasonic environment, and cause immunogenic death of cancer cells, a manner of tumor cell death that can trigger antitumor immune responses [37]. The overload of intracellular calcium under ultrasonic conditions leads to cell immunogenic death, which greatly promotes the process of antigen delivery to mature dendritic cells, and finally produces strong antitumor immunity.

At the same time, as verified by Jingyi an and Kaixiang Zhang et al., calcium can also enhance antitumor immunity in other ways. They prepared honeycomb calcium carbonate nanoparticles (OVA@CaCO_3_, denoted as HOCN) with ovalbumin (OVA) as the skeleton [14]. Firstly, their research found that the degradation behavior of HOCN in response to the microenvironment at the tumor site improved the low-pH environment, which inhibited the maturation of dendritic cells. Secondly, the calcium ions released after the nanoparticles were ingested by dendritic cells destroyed the autophagy inhibition conditions of dendritic cells (DCs). Autophagy of DCs can further activate T cells and eventually enhance antitumor immunity. Finally, after the tumor cells swallowed the nanoparticles, overloaded calcium ions in tumor cells promote the release of damage-associated molecular patterns (DAMPs) and the maturation of dendritic cells. This study provides us with a new research idea. Calcium-based inorganic nanoparticles can also regulate the calcium concentration in immune cells. At the same time, the calcium concentration in immune cells can also affect the related immune process, so as to promote antitumor immunity.

### 2.4. Intracellular Chloridion

**Figure 5:**
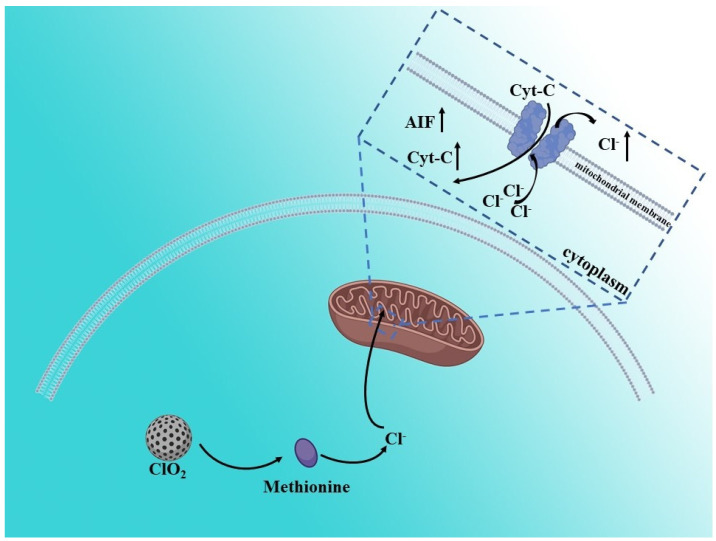
Schematic illustration of Cl^−^—induced antitumor therapy. The released Cl^−^ by ClO_2_, which can enter mitochondria through the voltage-dependent anion channel (VDAC), leads to mitochondrial damage and membrane potential decline, which further induce cell apoptosis.

In addition to cations, anion homeostasis in cells also has a great impact on cell life activities. The balance of normal anion concentration in cells provides a basis for maintaining cell morphology and function [38]. Compared with normal cells, cancer cells are more sensitive to anion homeostasis [38]. Therefore, interfering with anion homeostasis in cancer cells is also an effective means to achieve cancer treatment. Chloridion, as the most common anion in cells, has been widely studied. Synthetic anion transporters, a type of small molecular organic compound with transmembrane anion transport activity, can interfere with the homeostasis of cell anions, especially chloride ions, and trigger cell death [38]. Adil S. Aslam et al. proved that disturbed chloride ion concentration induced by an ion transporter in a cellular level affects endoplasmic reticulum (ER) stress by increasing Ca^2+^ concentration, and leads to apoptosis [39]. Similarly, Dong Un Lee et al. utilized cetrimonium bromide (CTAB: cationic quaternary amino group-based) gold nanorods to prove that the burst release of Cl^−^, as a result of lysosomal swelling by gold nanorods, induced a massive Ca^2+^ influx, which eventually promotes the apoptosis of cancer cells [40]. Meanwhile, Yuping Jiang et al. prepared a ClO_2_-loaded CaSiO_3_ nanoparticle which can produce large numbers of Cl^−^ by capturing methionine to disturb the balance of homeostasis in cancer cells (Figure 5). The released Cl^−^ by ClO_2_, which can enter the mitochondria through the voltage-dependent anion channel (VDAC), leads to mitochondrial damage and membrane potential decline, which further induce cell apoptosis [41]. In general, chloride ions directly or indirectly affect the ion homeostasis at different organelle levels in cells, including the endoplasmic reticulum, lysosome and mitochondria, thus affecting cell activity.

Similar to other ion interference treatment methods, chloride-based treatment often has better antitumor effects in combination with other treatment methods, such as starvation therapy. Therefore, this combination strategy is expected to be adopted to build more multifunctional platforms for in vivo treatment. Through the previous research, it can be found that ion interference therapy always needs to be combined with other treatment methods in order to achieve a better therapeutic effect.

Although there are few studies on the antitumor immunity of chloride ions, we can still expect that chloride-mediated apoptosis will have a positive effect on cancer immunotherapy.

### 2.5. Manganese, Copper, and Cobalt That Activate Biocatalysis

Enzymes are powerful catalysts to complete various chemical reactions related to life activities, such as metabolism, detoxification and biosynthesis [42]. Due to the high catalytic efficiency, some biological enzymes are used to treat various diseases, including inhibiting tumor growth. Glucose oxidase (GO_x_), which has the function of catalyzing the decomposition of glucose into H_2_O_2_, has been recognized as a “star” enzyme catalyst involved in cancer treatment [43]. Hanchun Yao et al. used collagenase, which can etch the dense extracellular matrix around tumor cells to remodel tumor microenvironment, so as to treat cancer more effectively [44]. However, due to the high synthesis cost and specific constraints of microenvironment where cells survive, biological protease used to treat diseases is difficult to popularize in the clinic.

With the development of nanotechnology, some ions with a catalytic function were developed to replace enzymes, and inorganic nanoparticles were used as carriers to inhibit tumor proliferation. Iron-based nanozymes (INs) are one of the earliest inorganic nanomaterials with exploitable catalytic behaviors [45]. Fe^2+^ can catalyze the reaction of H_2_O_2_ into the highly toxic ⋅OH, which can inhibit tumor proliferation [46]. Mn^2+^, Cu^+^ and Co^2^^+^, which are delivered by inorganic nanoparticles into tumor cells, can also produce similar catalytic reactions as Fe^2+^, so they are also used for ion interference therapy.

**Figure 6:**
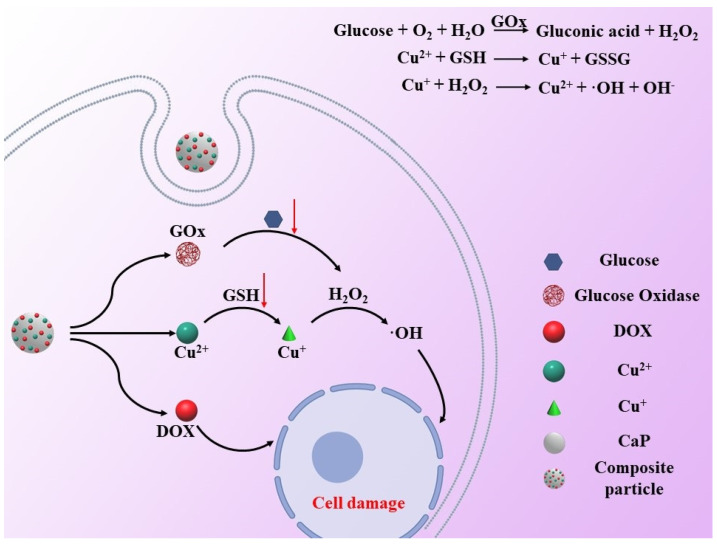
Schematic illustration of Cu^+^—induced antitumor therapy. The released Cu^2+^ with the degradation of calcium phosphate in tumor cells can react with glutathione to form the Fenton agent Cu^+^, which further triggers the H_2_O_2_ to generate ⋅OH to enhance the antitumor effect.

Lian-Hua Fu et al. constructed a Cu^2+^-doped calcium phosphate nanoparticles. The released Cu^2+^ with the degradation of calcium phosphate in tumor cells can react with glutathione to form the Fenton agent Cu^+^, which further triggers the H_2_O_2_ to generate ⋅OH to enhance the antitumor effect (Figure 6) [47]. They also proved that Mn^2+^-mediated Fenton-like reaction enhanced chemotherapy by using biodegradable manganese-doped calcium phosphate [43]. Hanjing Kong et al. synthesized fine CaO_2_ nanoparticles with Cu–ferrocene molecules at the surface (CaO_2_/Cu–ferrocene). Under an acidic condition, the particles release Ca^2+^ ions and H_2_O_2_ in a rapid fashion. The Fenton reaction between Cu^+^ (from Cu–ferrocene) and H_2_O_2_ induced significant in vitro and in vivo antitumor phenomena by producing a large amount of ⋅OH [48]. Shutao Gao et al. also proved that Co^2+^ is also a Fenton agent that can enhance the antitumor effect by using a ZIF-67 coated CaO_2_ nanoparticle (CaO_2_@ZIF-67) [49]. The nanoparticle is broken down in the weakly acidic environment within tumors to rapidly release the Fenton-like catalyst Co^2+^. As with other Fenton reagents, Co^2+^ reacts with H_2_O_2_, which was released by degraded CaO_2_ to produce a large number of ⋅OH, which eventually leads to the death of tumor cells.

Compared with other forms of ion interference therapy, this ion interference therapy that activates the biocatalysis process does not depend on other traditional cancer treatments. A single ion treatment can produce good therapeutic effects in vitro and in vivo. Therefore, this treatment method is more reliable and has more potential to achieve practical clinical applications. For this treatment, it is worth noting that they all rely on H_2_O_2_ to start the tumor killing program, and Ca_2_O is both a good donor of H_2_O_2_ and Ca^2+^. Therefore, the combination of calcicoptosis and manganese, copper, and cobalt ion interference therapy using Ca_2_O may be a more effective anti-tumor plan. In addition, there are also some new developments in the field of immunotherapy. Mengyu Chang et al. conducted further research by using Cu_2_O@CaCO_3_ modified with hyaluronic acid. They found that the oxidative stress caused by Cu^+^ from Cu_2_O@CaCO_3_ nanocomposites can efficiently reprogram the macrophages from the M2 phenotype to the M1 phenotype and initiate a vaccine-like immune effect after primary tumor removal, which further induces an immune-favorable tumor microenvironment and intense immune responses for anti-CD47 antibody to simultaneously inhibit distant metastasis and recurrence by immunotherapy [36]. It can be predicted that the combination of ion interference therapy and immunotherapy will have a more significant antitumor effect.

## 3. Conclusions and Perspectives

In summary, once the homeostasis of some common ions in cells is abnormal, such as specific accumulation or reduction, it will affect the normal physiological activities of cells. The treatment of inhibiting cancer cell proliferation by artificially interfering with the balance of ion homeostasis in tumor cells is called ion interference therapy. Compared with small molecular ion transport carriers with limited transport efficiency, inorganic nanoparticles that can release or absorb a large number of ions more quickly have become the best medium for ion interference therapy (Table 1). The homeostasis imbalance of different ions mediated by different inorganic nanoparticles induces the death of cancer cells by different principles. Several different principles of different ions on interference therapy are summarized as follows: (a) a great quantity of H^+^ released from damaged lysosomes, caused by the rapid degradation or expansion of nanoparticles acidify the cytoplasmic environment and induce cancer cell death; (b) the imbalance of a large amount of sodium or potassium concentration gradient, caused by the transportation of sodium or potassium nano-inorganic salt into cancer cells, leads to the change in cell osmotic pressure, makes the cells swell, and finally induces pyroptosis; (c) calcium overload in tumor cells mediated by calcium-based inorganic nanoparticles damages the normal function of mitochondria and further induces apoptosis, which is called calcicoptosis; (d) The specific accumulation of chloride ions in the mitochondria of tumor cells leads to the decrease in mitochondrial membrane potential, the release of mitochondrial contents and the induction of apoptosis; (e) some Fenton agents produced by the degradation of inorganic nanoparticles, including Cu^+^, Mn^2+^ and Mn^2+^, can trigger the H_2_O_2_ to generate ⋅OH to enhance the anti-tumor effect. On the other hand, apoptosis and pyroptosis are both forms of programmed cell death, mediated by ion interference therapy, which enhances the effects of antitumor immunity. Among these nanoparticles, Ca^2+^ promotes the effects of antitumor immunity by destroying the autophagy inhibition conditions of dendritic cells, a special mechanism compared with others.

Compared with traditional tumor treatment, such as chemotherapy and radiotherapy, ion interference treatment based on inorganic nanoparticles shows higher biocompatibility and further therapeutic effects in vivo due to the lower tolerance of tumor cells to abnormal ion concentration compared to normal cells. Although ion interference therapy is a practical method, and different ions have different action mechanisms, it still has many aspects worthy of in-depth exploration.

Through previous research, it can be found that ion interference therapy always needs to be combined with other treatment methods in order to achieve better therapeutic effects. Ion interference therapy alone does not seem to produce obvious antitumor effects, such as for Ca^2+^, which may be related to the degradation efficiency of inorganic nanoparticles, the in vivo circulation time, the ability to release or adsorb ions, and the metabolic rate of tumor cells. Therefore, finding ways to improve the performance of inorganic nanoparticles based on the above aspects is the next research direction that needs to be focused on. At the same time, it should also be considered whether there is synergy between different ion therapy methods to promote antitumor effects, such as Cu^+^ and Ca^2+^. The combination of various types of ion interference therapy also has broad development prospects. In addition, the combination of ion interference therapy and immunotherapy cannot be ignored. The effects of different ions on immune cells and the process of antitumor immunity need to be further studied. Additionally, the use of new composite inorganic nanoparticles combined with ion interference therapy and immunotherapy will provide a promising strategy for enhancing antitumor immunity.

## Figures and Tables

**Table 1:** The classification of inorganic nanomaterials for IIT.

Inorganic Nanoparticles	Interfering Ions	Mechanism	Reference
CaCO_3_ NPs	H^+^	Intracellular pH	[20]
NaCl NPs	Na^+^	Osmotic pressure	[11]
Na_2_S_2_O_8_ NPs	Na^+^	Osmotic pressure	[27]
K_3_ZrF_7_:Yb/Er	K^+^, [ZrF_7_]^3−^	Osmotic pressure	[28]
SH-CaO_2_ NPs	Ca^2+^	Calcicoptosis	[8]
CaP NPs	Ca^2+^	Calcicoptosis	[34]
Amorphous calcium carbonate (ACC NPs)	Ca^2+^	Calcicoptosis	[7]
CaCO_3_@COF-BODIPY-2I@GAG	Ca^2+^	Calcicoptosis	[35]
CaBPs	Ca^2+^	Calcicoptosis	[4]
Cu_2_O@CaCO_3_	Cu^+^, Ca^2+^	Biocatalysis, calcicoptosis	[36]
CaCO_3_@PEG	Ca^2+^	Calcicoptosis	[37]
OVA@CaCO_3_	Ca^2+^	Calcicoptosis	[14]
ClO_2_	Cl^−^	Organelle stress	[41]
CaO_2_/Cu–ferrocene	Ca^2+^, Cu^+^	Calcicoptosis, biocatalysis	[48]
Cu-CaP NPs	Cu^2+^	Biocatalysis	[47]
Mn-CaP NPs	Mn^2+^	Biocatalysis	[43]
CaO_2_@ZIF-67	Co^2+^	Biocatalysis	[49]

## Data Availability

Not applicable.
